# Members of *Gammaproteobacteria* as indicator species of healthy banana plants on *Fusarium* wilt-infested fields in Central America

**DOI:** 10.1038/srep45318

**Published:** 2017-03-27

**Authors:** Martina Köberl, Miguel Dita, Alfonso Martinuz, Charles Staver, Gabriele Berg

**Affiliations:** 1 Graz University of Technology, Institute of Environmental Biotechnology, Austria; 2 Brazilian Agricultural Research Corporation – Embrapa, Brasília, Brazil; 3 Bioversity International, Turrialba, Costa Rica; 4 Bioversity International, Montpellier, France

## Abstract

Culminating in the 1950’s, bananas, the world’s most extensive perennial monoculture, suffered one of the most devastating disease epidemics in history. In Latin America and the Caribbean, *Fusarium* wilt (FW) caused by the soil-borne fungus *Fusarium oxysporum* f. sp. *cubense* (FOC), forced the abandonment of the Gros Michel-based export banana industry. Comparative microbiome analyses performed between healthy and diseased Gros Michel plants on FW-infested farms in Nicaragua and Costa Rica revealed significant shifts in the gammaproteobacterial microbiome. Although we found substantial differences in the banana microbiome between both countries and a higher impact of FOC on farms in Costa Rica than in Nicaragua, the composition especially in the endophytic microhabitats was similar and the general microbiome response to FW followed similar rules. Gammaproteobacterial diversity and community members were identified as potential health indicators. Healthy plants revealed an increase in potentially plant-beneficial *Pseudomonas* and *Stenotrophomonas*, while diseased plants showed a preferential occurrence of *Enterobacteriaceae* known for their plant-degrading capacity. Significantly higher microbial rhizosphere diversity found in healthy plants could be indicative of pathogen suppression events preventing or minimizing disease expression. This first study examining banana microbiome shifts caused by FW under natural field conditions opens new perspectives for its biological control.

Bananas are the world’s most important fruit in terms of production volume and trade^1^. Only 3% of the global banana production (>135 million tons per year) is traded internationally^2^,^3^, indicating their importance for food security. Latin America and the Caribbean (LAC) grow 28% of the global production and nearly 20 million tons are consumed locally. Seven countries of the region are in the top 10 banana exporting nations. The region also produces 72% of exported plantains^4^. Pests and diseases, frequently referred as one of the most limiting factors for banana production worldwide, are also a major problem in LAC^4^,^5^. In fact, LAC experienced one of the most devastating plant disease epidemics in history. In the 1950 s, *Fusarium* wilt (FW) of banana, caused by the soil-borne fungus *Fusarium oxysporum* f. sp. *cubense* (FOC), forced the abandonment of the Gros Michel banana industry^6^,^7^. *Fusarium* wilt, also known as Panama disease, is particularly devastating, because FOC produces chlamydospores, survival structures that remain in the soil for decades in the absence of suitable hosts. The effects of FOC race 1 (R1) were overcome by a shift to resistant Cavendish cultivars which are currently the source of 99% of banana exports^1^,^2^. However, since early 1990 s, severe FOC infestations, caused by the new variant called as tropical race 4 (FOC TR4) are being recorded on Cavendish plantations^3^,^6^,^7^,^8^. FOC TR4 was restricted to Asia and northern Australia for about 20 years, but has been reported recently in Jordan (2012), Oman (2012), Mozambique (2013), Lebanon (2015), Pakistan (2015) and Queensland, Australia (2015). Even with expansion of Cavendish varieties, millions smallholders in LAC continued depending on FOC R1-susceptible varieties, which are preferred on local markets. Aiming to reduce the FW impact, some of these varieties are grown in agroforestry systems (AFS), mainly associated with *Coffea* and *Fabaceae* trees^9^. Although FW epidemics in AFS might be generally lower compared with intensive monocultures, farmers still face the impact of the disease, which often limits both expansion and increased farmer’s income. This requires new ways to control the pathogen efficiently.

Currently, the use of resistant varieties is the only known option for producing bananas on FOC-infested soils. However, resistant varieties do not always meet consumer’s preferences or are not available as is the case of FOC TR4^10^,^11^. In addition, resistant varieties are considered temporary solutions as resistance “break down” may occur at any time^12^. Therefore, integrated disease management strategies are needed aiming both to reduce the impact of the disease on susceptible varieties and increase the durability of resistant varieties. In this scenario, banana and soil microbiota are hypothesized to play an important role^13^,^14^ as already shown in other agricultural systems^15^,^16^. Recently, we showed that AFS lead to shifts within the gammaproteobacterial microbiome of banana plants cultivated in Central America^9^,^17^. *Gammaproteobacteria* have been identified as crucial part within other plant microbiomes^18^,^19^. Although little is known about the plant and soil microbiome profiles and potential relationships to FW infestation in banana, we hypothesize a substantial response of *Gammaproteobacteria*.

In the present study, we analyzed the relation of the gammaproteobacterial microbiome of Gros Michel bananas and FW (FOC R1) by comparing FW-infested and healthy banana plants under AFS in Costa Rica and Nicaragua. In each country, samples of banana roots, pseudostem, leaves and rhizosphere soil were analyzed from three different farms using gammaproteobacterial 16S rRNA gene profiling. Our results suggest that some plant-associated members of *Gammaproteobacteria* might be involved in the process that keeps banana plants FW-free in FOC-infected areas. In addition, we show for the first time how FOC infection affects the banana plant microbiome under natural field conditions.

## Results

### Richness and diversity of the gammaproteobacterial community

A barcoded 16S rRNA gene amplicon sequencing approach based on Illumina MiSeq sequencing of the gammaproteobacterial microbiota associated to the rhizosphere, endorhiza, pseudostem and leaves of healthy and FOC-infected banana plants grown in Nicaragua and Costa Rica yielded in 4,473,541 gammaproteobacterial quality sequences with a read length ≥200 nucleotides, between 4,822 and 111,332 quality reads per sample. Averaged rarefaction analyses of the sequencing libraries at a genetic dissimilarity level of 3% are depicted in Fig. S1. Comparisons of observed OTUs with their estimated richness by the Chao1 index revealed coverage between 97.5 and 39.0% per sample at order level (Table S1). The sequencing efforts at genus and species level reached 89.2–32.5% and 69.1–27.6%, respectively. The computed Shannon indices of diversity (H’) ranged from 7.35 to 0.26 at a genetic distance of 3% (Table S1). In asymptomatic plants (Fig. S2), the highest values were observed for endorhiza samples from Costa Rica (5.97 on average ±0.83 confidence), showing no significant difference (p ≤ 0.05, Tukey-HSD post hoc test) to the rhizosphere soil from Costa Rica (4.40 ± 1.16) but to both above-ground microenvironments, leaves (3.33 ± 0.72) and pseudostem (3.06 ± 0.46). Within samples from Nicaragua, the highest gammaproteobacterial diversity was found for the rhizosphere soil (5.27 ± 0.86), but as in Costa Rica it was not significantly different from the endorhiza (4.38 ± 1.01). Significantly lower Shannon values than in the rhizosphere were detected for pseudostem (2.57 ± 0.43) and leaf samples (2.54 ± 0.48). Between the same microenvironments of banana plants from the two countries, no significant differences were observed. No influence of FW infestation on the gammaproteobacterial diversity of the different microenvironments was observed, except the rhizosphere of bananas cultivated in Costa Rica, where healthy plants showed a significantly higher diversity (p = 0.025, independent samples *t*-test) (Fig. S2).

### Taxonomic composition

The taxonomic composition of the gammaproteobacterial banana microbiome inhabiting the individual microenvironments of healthy Gros Michel plants cultivated in Nicaragua and Costa Rica was analyzed in detail in our previous study comparing different agroforestry conditions^9^. In the present study encompassing samples from healthy and FW-infested plants, nearly all quality sequences could be assigned below the class level. Over all banana-associated communities, high abundances of *Pseudomonadales, Enterobacteriales, Xanthomonadales* and *Legionellales* were found (Fig. S3). At lower taxonomic levels, *Pseudomonadales* could be assigned to *Pseudomonadaceae* (genus *Pseudomonas*) and *Moraxellaceae* (genera *Acinetobacter, Enhydrobacter* and *Perlucidibaca*). In general, samples from Nicaragua were highly dominated by *Pseudomonadaceae* while samples from Costa Rica revealed a high abundance of *Moraxellaceae* (Fig. S3). The enterobacterial fraction was dominated by *Erwinia* with lower abundances of *Enterobacter, Serratia* and *Citrobacter. Xanthomonadales* sequences could be assigned to different *Xanthomonadaceae (Stenotrophomonas, Pseudoxanthomonas, Luteimonas, Dokdonella, Rhodanobacter* and *Luteibacter*) and *Sinobacteraceae (Steroidobacter* and *Nevskia*). *Legionellales* could be divided into the families *Coxiellaceae (Aquicella* and *Rickettsiella*) and *Legionellaceae (Legionella, Tatlockia*). Further genera identified for taxonomic groups with a relative abundance over 1% per sample belonged to *Alteromonadales (Cellvibrio* and *Rheinheimera*) and *Oceanospirillales (Halomonas*). Taxonomic groups exclusively found in FOC-infected plants could be affiliated to the families *Thiotrichaceae, Alcanivoracaceae, Succinivibrionaceae* and *Chromatiaceae*. Members of the family *Pseudoalteromonadaceae* were exclusively detected in healthy plants.

### Impact of *Fusarium* wilt on the gammaproteobacterial banana microbiome

**Figure 1:**
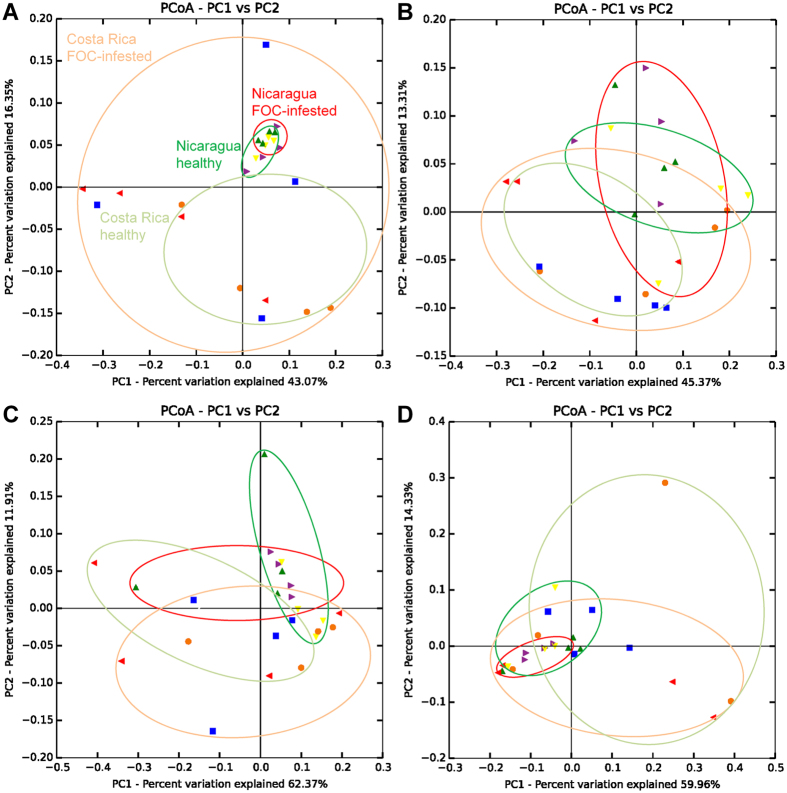
Principal coordinate analysis (PCoA) plots of the gammaproteobacterial communities inhabiting rhizosphere soil (**A**), endorhiza (**B**), pseudostem (**C**), and leaves (**D**) of banana plants infected by *Fusarium oxysporum* f. sp. *cubense* (FOC) in comparison to healthy plants. PCoA biplots are based on weighted UniFrac distances of gammaproteobacterial 16 S rRNA gene amplicon libraries jackknife-supported by ten replicates. The plots indicate grouping of samples by country separating samples from healthy (green) and infested (red) plants, whereby different colored symbols point to individual farms. Statistical comparisons of healthy and FOC-infected plants based on weighted UniFrac distances are shown in the Supplementary Information (Table S2).

Principal coordinate analyses of the gammaproteobacterial communities colonizing the banana plant in its different microenvironments were performed in order to compare healthy and FOC-infected plants (Fig. 1). Endorhiza and pseudostem samples exhibited similar scatters for both countries with a relatively large overlap. In contrast, leaf and especially rhizosphere samples from Nicaragua revealed a much lower scattering in comparison to samples from Costa Rica. Although, the PCoA plots based on weighted UniFrac distances indicated slight differences in the gammaproteobacterial community patterns of healthy and infested plants, no significant differences (p ≤ 0.05, adonis test) could be calculated (Table S2). The biggest difference between healthy and FOC-infected state was observed for the rhizosphere soil of plants cultivated in Costa Rica (p = 0.09, adonis test).

**Figure 2:**
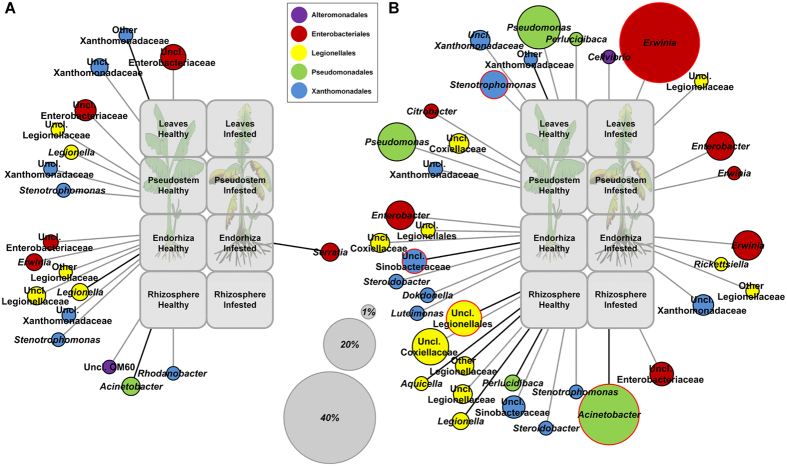
Profile clustering network analyses depicting the impact of infestation with *Fusarium oxysporum* f. sp. *cubense* on the gammaproteobacterial microbiome of banana plants cultivated in Nicaragua (**A**) and Costa Rica (**B**). The relative abundances of OTUs at a dissimilarity level of 3% summarized at genus level with a mean read change between healthy and infested state of more than 0.5% of the data set were used. If the ratio of relative mean abundances exceeded 1.5, the taxa were regarded as altered and assigned to the respective profile. Only those taxonomic groups that featured the same pattern in at least two farms of the respective country are shown (grey connection lines: same pattern in two farms; black connection lines: same pattern in all three farms). Node sizes correspond to the abundance change between healthy and infested state; nodes matching to changes of 1%, 20% and 40% of the data set were added as reference points. Significant differences (p ≤ 0.05, Metastats) are indicated by red node borders.

However, profile clustering network analyses confirmed that individual taxonomic groups were not evenly distributed in healthy and FOC-infected banana plants (Fig. 2). The gammaproteobacterial communities of plants were more sensitive to the FOC infection in Costa Rica than in Nicaragua (Fig. 2). In both countries, more taxa appeared in considerably higher abundances in the healthy state of the plant, with the exception of *Enterobacteriaceae* which mostly revealed higher presence in *Fusarium*-infected banana plants, especially in those from Costa Rica. In general, Costa Rica’s infested plants revealed higher numbers of different *Enterobacteriaceae (Erwinia, Enterobacter* and other unclassified *Enterobacteriaceae*) in comparison to healthy plants. The genus *Erwinia* showed the highest abundance values, especially in leaves (p ≤ 0.05, Metastats). Conversely, samples from healthy plants from Costa Rica showed higher abundances of several potential pathogen-suppressing gammaproteobacterial genera, such as *Stenotrophomonas* and *Pseudomonas*. In addition, higher numbers of distinct *Xanthomonadales (Sinobacteraceae* and *Xanthomonadaceae*) and *Legionellales (Coxiellaceae* and *Legionellaceae*) were present in healthy plants in Costa Rica (Fig. 2B). *Fusarium* wilt did not cause significant impact on the gammaproteobacterial communities from Nicaragua when compared with Costa Rica. However, healthy plants from Nicaragua also unveiled higher abundances of potential plant-beneficial *Stenotrophomonas* colonizing the endosphere of the banana plant (pseudostem and endorhiza). On all three Nicaraguan farms, *Acinetobacter* was observed in higher abundances in the rhizosphere of healthy plants (Fig. 2A). This genus was also found in significantly higher counts in the rhizosphere of Costa Rica’s, but in the FOC-infected plants. However, while in Nicaragua *A. rhizosphaerae* predominated, in Costa Rica *A. johnsonii* was the main species found.

### The healthy rhizosphere core microbiome and its co-occurrence relationships

**Figure 3:**
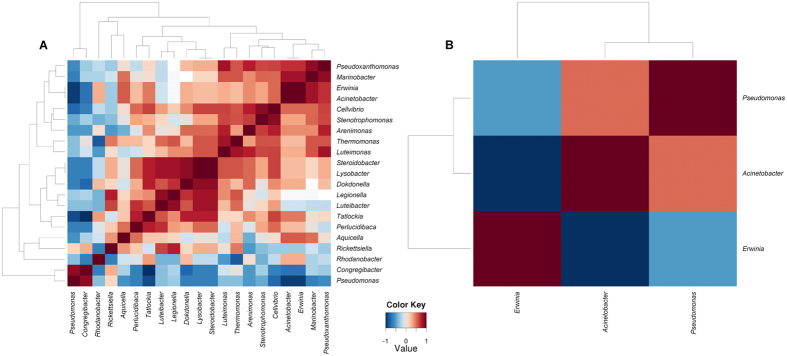
Spearman rank correlation structure between genera abundances of the rhizosphere core microbiome of healthy banana plants cultivated in Nicaragua (**A**) and Costa Rica (**B**). Core OTUs at a dissimilarity level of 3% with the same taxonomic assignment at genus level were combined, and unassigned reads were excluded from the dataset.

The vast majority (90.6% of reads and 17.9% of OTUs_0.03_) of the gammaproteobacterial rhizosphere community associated with healthy banana plants cultivated in Nicaragua could be assigned to the core microbiome, which was defined as those OTUs that were present in at least 50% of the respective samples. The rhizosphere core microbiome of plants from Costa Rica comprised only 30.9% of reads and 1.1% of OTUs_0.03_. Consequently, the diversity within the rhizosphere core microbiome of Nicaragua’s plants was higher and OTUs could be affiliated to 21 distinct gammaproteobacterial genera, while the core OTUs of Costa Rica’s plants were classified to only 3 different genera (*Acinetobacter, Erwinia* and *Pseudomonas*) (Fig. 3). In the rhizosphere core microbiome of Nicaragua, the genera *Pseudomonas, Acinetobacter, Legionella, Rhodanobacter, Steroidobacter, Erwinia, Dokdonella* and *Luteimonas* were found in a relative abundance over 0.1%.

In general, Spearman’s rank correlations between occurrence patterns of the gammaproteobacterial core genera were dominated by positive correlations (Fig. 3). The strongest positive co-occurrence relationships in the rhizosphere core microbiome of Nicaragua were identified for *Steroidobacter* and *Lysobacter*, and for *Erwinia* and *Acinetobacter*. Strongest negative correlations in Nicaragua were found between *Pseudomonas* and *Erwinia, Pseudomonas* and *Acinetobacter*, and between *Congregibacter* and *Tatlockia* (Fig. 3A). In Costa Rica, an indirect correlation was observed between *Erwinia* and *Acinetobacter* (Fig. 3B).

## Discussion

In this study, we deciphered the microbiome associated with healthy and FW-diseased Gros Michel bananas in two countries from Central America to understand its importance for the health status. Our results revealed for the first time i) the interaction of FW with the banana microbiome under field conditions, and ii) uncovered gammaproteobacterial diversity and identified community members as potential health indicators. Interestingly, although we found substantial differences in the banana microbiome between both studied countries, the general response to FW followed similar rules. The resistance of a microbial community to invasion of pathogens is linked to its level of diversity^20^,^21^,^22^. In addition, it has been reported that plant pathogens can be responsible for drastic shifts and dysbiosis within the plant-associated microbiome^23^ as well as for long-term dysbiosis within the soil microbiome, resulting in infested soils that are unable to be replanted with susceptible plants for a long time^14^,^24^. In our study, we found both, dysbiosis in the FOC-infested banana microbiomes and a relationship between disease and microbial diversity.

In terms of diversity, our results showed that FOC significantly influenced the gammaproteobacterial diversity in the banana rhizosphere of plants grown in Costa Rica, which was significantly reduced in FOC-infected plants. On average, all investigated plant microenvironments (rhizosphere, endorhiza, pseudostem and leaves) achieved lower diversity measurements in the infested than in the healthy state. FOC infection is characterized by an aggressive colonization of vascular plant tissues^25^, which could explain this reduction in the overall diversity. The significant loss in diversity found for the rhizosphere soil of diseased plants in Costa Rica might be associated to a higher FOC density in the soil of those farms. Although the sampled plants had similar disease levels, in general, levels of FW incidence in Costa Rica were higher than in Nicaraguan farms. Lian *et al*.^13^ uncovered an increased bacterial diversity in tissue-culture plantlets at early stages of FOC infection compared to un-inoculated control plantlets under greenhouse conditions. This study^13^ used banana tissue-culture plantlets artificially treated with a crude homogenate of roots from healthy banana plants that were later (3 weeks after) inoculated with FOC. Under these conditions, a higher diversity of endophytic bacteria communities was observed compared with un-infected plants, suggesting that at early stages of FOC infection banana plants recruit bacteria as a defense mechanism. The present study was performed on banana plants grown under natural field conditions and each sampled infested plant showed class 2 disease symptoms (advanced yellowing of older leaves progressing to intermediate and younger leaves), which enabled a consistent comparison between healthy and FW-diseased plants. The latent contact with the soil-borne pathogen potentially encouraged attraction of plant-beneficial antagonists and consequently resulted in an increase in community diversity. In accordance with this hypothesis, Lian *et al*.^13^ assumed that the FW may be inhibited by a diverse bacterial community maintaining the pathogen spores below the level that is required for expression of pathogenicity. Similar findings were reported with avocado trees that were infected with the pathogen *Phytophthora cinnamomi*^26^. Further studies, including more complete population microbiology are necessary to understand this behavior, but defending soil and plant microbiota will definitely be key factors.

In terms of community structure, the rhizosphere soil from Costa Rica revealed the most considerable differences between healthy and infested plants. In FW-infested banana plants from Costa Rica, certain *Enterobacteriaceae (Erwinia, Enterobacter*, and other unclassified genera) were increased considerably, especially in above-ground plant parts. A recent study of the lettuce (*Lactuca sativa* L.) microbiome also revealed a preferential occurrence of enterics in the phyllosphere and an additional enhancement in bottom rot (*Rhizoctonia solani* Kühn)-infested plants^19^. However, while enriched enterobacterial taxa in diseased lettuce plants could primarily be affiliated to *Enterobacter, Erwinia* was the most affected genus in FOC-infected banana plants. In contrast to *Enterobacter*, which comprises several opportunistic human pathogenic strains (e.g. *E. aerogenes, E. cloacae*)^23^, *Erwinia* is mainly known as plant pathogen (e.g. *E. amylovora, E. tracheiphila*)^27^,^28^. Although the function of these potentially pathogenic bacteria in FOC-infested banana plants is unclear, research especially on multi-pathogen diseases showed that bacteria can use the mycelium of fungal pathogens for translocation^29^. Pathogenic fungi like FOC in banana may eventually pave the way for rapid access of pathogenic bacteria to the plant tissue. The increased weakness of infected plants may also contribute positively with this phenomenon. However, no relationship between FW and *Erwinia*-associated diseases has been reported so far in banana. Most of the identified banana-colonizing genera could not be safely assigned to species level. However, the *Acinetobacter* community colonizing the rhizosphere of healthy banana plants from Nicaragua was highly dominated by *A. rhizosphaerae*, while the FOC-infested banana rhizosphere from Costa Rica were mainly colonized by *A. johnsonii*. These differences confirm that shifting in microbial communities can reach species and even strain level^30^. Both identified *Acinetobacter* species are ubiquitous and have been found in the rhizospheres of plants before. However, while *A. rhizosphaerae* is known as phosphate-solubilizing plant growth-promoting rhizobacterium^31^,^32^ and has been associated with bioremediation^33^, *A. johnsonii* – despite generally recognized as non-pathogenic – is often found in hospital environments and was identified to have several homologous genes to known virulent proteins of *A. baumannii*, a global nosocomial pathogen^34^.

Our results open new perspectives to biologically control FW in banana. For example, comparative analyses between infested and healthy plants revealed unclassified plant-associated species of *Stenotrophomonas* and *Pseudomonas* as potential health indicators, which can be used as biological control agents. These two gammaproteobacterial genera were also found in increased abundances in the microbiome of Gros Michel bananas grown under legume-based agroforestry conditions, where a lower FW incidence was noted in comparison to banana monocultures^9^,^17^. Both genera are known to comprise some plant-beneficial species: For example, the species *Stenotrophomonas rhizophila* has become a model bacterium for a rhizosphere- and phylloplane-competent, salt- tolerant plant growth promoter and stress protecting agent^35^,^36^,^37^,^38^. Members of the genus *Pseudomonas*, such as *P. fluorescens* or *P. putida*, are besides *Bacillus* spp. probably the most prominent plant growth-promoting bacteria, which have been extensively utilized and studied over the last decades^39^. Both genera, *Pseudomonas* and *Stenotrophomonas*, have already been mentioned in association with the suppression of *Fusarium oxysporum*, for example of the f. sp. *ciceris* on chickpea, *Cicer arietinum* L.^40^. Future biocontrol studies on FW on bananas should consider applications of antagonistic, plant growth-promoting *Pseudomonas* and *Stenotrophomonas* spp. and carefully investigate their impact on non-target (micro)organisms and soil health. In addition to some potentially health-indicating taxa, the aforementioned increased microbial rhizosphere diversity, which is substantially correlated with a low incidence of pathogen outbreaks^16^,^20^,^21^, could possibly counteract FW in banana. Accordingly, all management practices with a potential positive impact on microbial soil diversity, such as intercropping, agroforestry or organic soil amendments, can therefore be recommended for an improved plant health and banana crop performance.

## Methods

### Experimental design and sampling procedure

Samples were taken in November 2012 from *Musa acuminata* Colla (AAA genome) cultivar Gros Michel in Nicaragua, department Jinotega, and in Costa Rica, canton Turrialba. In each country, samples of banana roots, pseudostem, leaves and rhizosphere soil were collected from three different farms affected by *Fusarium* wilt (FW). On every sampling farm, banana plants are cultivated intercropped with *Coffea* and under legume-based agroforestry conditions. The predominant trees were *Inga* spp. in Nicaragua and *Erythrina poeppigiana* in Costa Rica. At the time of sampling, each site had been under shaded coffee production for at least 30 years. The exact locations of the farms are given in Köberl *et al*.^9^, where the impact of agroforestry was investigated. In each farm, two areas, healthy (no symptoms of FW) and FW-infested, were identified. For each microenvironment, two replicate composite samples consisting of sub-samples from five plants were taken. All the plants sampled were at the same physiological stage and sampled plants affected by FW showed similar disease severity values (class 2 disease symptoms: advanced yellowing of older leaves progressing to intermediate and younger leaves).

### Total community DNA isolation

For extraction of metagenomic DNA from the rhizosphere, 2 g of rhizospheric soil were mixed with 15 ml of 0.85% NaCl for 10 sec on the vortex. To isolate total community DNA from the endorhiza, 5 g of roots were surface-sterilized with 4% NaOCl for 5 min. Afterwards, roots were washed three times with sterile distilled water and transferred to sterile Whirl-Pak bags (Nasco, Fort Atkinson, WI, USA), then 10 ml of 0.85% NaCl were added and the surface-sterilized roots were homogenized using mortar and pestle. Pseudostem samples (5 g) were washed with sterile distilled water, transferred to Whirl-Pak bags, and after 10 ml of 0.85% NaCl were added, homogenized with mortar and pestle. From phyllosphere samples, 5 g of leaves were washed three times with sterile distilled water, before homogenization with 10 ml of 0.85% NaCl. From the liquid parts, 4 ml were centrifuged at high speed (16,000 × g, 4 °C) for 20 min and resulting pellets were stored at −70 °C. Total community DNA was extracted using the FastDNA SPIN Kit for Soil (MP Biomedicals, Solon, OH, USA) according to the manufacturer’s protocol. Metagenomic DNA samples were encoded using abbreviations indicating: (1) country (N− = Nicaragua, C− = Costa Rica), (2) microenvironment (S = rhizosphere soil, Re = endorhiza, Ps = pseudostem, L = leaves), (3) farm (1–3 in each country), (4) status of infestation with *Fusarium oxysporum* f. sp. *cubense* race 1 (− = healthy, + = infested), and (5) independent replicate sample (1, 2).

### Gammaproteobacterial 16S rRNA gene profiling by Illumina MiSeq sequencing

For a deep-sequencing analysis of the banana-associated *Gammaproteobacteria* community, the hypervariable V4 region of the 16 S rRNA gene was amplified in a nested PCR approach with the *Gammaproteobacteria*-specific primer pair Gamma395f/Gamma871r^41^ and the universal primer pair 515 f/806r^42^, which carried sample-specific tags. The reaction mixture for the first PCR (20 μl) contained 1 × Taq&Go (MP Biomedicals, Eschwege, Germany), 2 mM MgCl_2_, 0.1 μM of each primer and 1 μl (~10 ng) of template DNA dilution (96 °C, 4 min; 30 cycles of 96 °C, 1 min; 54 °C, 1 min; 74 °C, 1 min; and elongation at 74 °C, 10 min). The second PCR (30 μl) was performed by using 1 × Taq&Go, 0.2 μM of each primer and 1,2 μl from dilutions (1:1000) of the first PCR mixtures (94 °C, 3 min; 32 cycles of 94 °C, 45 sections; 60 °C, 1 min; 72 °C, 18 sec; and elongation at 72 °C, 10 min). PCR products of three independent reactions were pooled in equal volumes and purified by employing the Wizard SV Gel and PCR Clean-Up System (Promega, Madison, WI, USA). Amplicon libraries were generated and sequenced by a paired-end approach using the Illumina MiSeq platform (Eurofins Genomics, Ebersberg, Germany). The nucleotide sequences are available in the European Nucleotide Archive (www.ebi.ac.uk/ena) under the BioProject accession number PRJEB12550.

Data were analyzed using the open source software package QIIME 1.8^43^. Sequencing reads with more than three consecutive low quality base calls (Phred quality score ≤25) were truncated at the position where their quality began to drop, and only reads with >75% consecutive high quality base calls, without any ambiguous characters, and longer than 200 nucleotides in length were retained for further analyses. All quality sequences were adjusted in the same orientation and clustered into operational taxonomic units (OTUs) with uclust^44^, using 3%, 5% and 10% dissimilarity thresholds. From each OTU the most abundant sequence was selected as the representative one, and the taxonomy of the representative set was assigned with the uclust-based consensus taxonomy assigner using an 80% confidence threshold. The representative sequence set was aligned with PyNAST^45^. Based on a check with ChimeraSlayer, potential chimeric sequences were discarded. OTU tables at the different dissimilarity levels were constructed, and OTUs not assigned to the class of *Gammaproteobacteria* and singletons were removed from the dataset. For alpha and beta diversity analyses, OTU tables were rarefied at 4,820 reads. Diversity indices Shannon^46^ and Chao1^47^ were determined based on the normalized clustering data. Significant differences were calculated with PASW Statistics 18 (SPSS Inc., Chicago, IL, USA) using Tukey-HSD and Games-Howell post hoc tests, depending on the homogeneity of variances, and the independent samples *t*-test for differences between healthy and *Fusarium* wilt-infested plants. Beta diversity was analyzed based on weighted UniFrac distances^48^ and ten jackknife replicates of the total rarefied datasets. Statistical analyses were performed using the adonis test with 999 permutations.

### Network analyses and correlation patterns

Profile clustering network analyses were performed to highlight single taxonomic groups corresponding to genus level (OTUs at a dissimilarity level of 3% summarized at taxonomic level 6) with considerable differences between healthy and infested state. The network analyses were carried out with taxa exhibiting a mean read change of more than 0.5% of the data set. If the ratio of relative mean abundances exceeded 1.5, the taxa were regarded as altered and assigned to the respective profile. Only taxonomic groups featuring the same pattern in at least two farms of the respective country were considered. Visualization of the networks was carried out using Cytoscape 2.8.3^49^. Significant differences were ascertained with Metastats^50^, where p values were computed using a combination of the nonparametric *t*-test, exact Fisher’s test, and the false discovery rate with 10^3^ permutations. For the determination of the rhizosphere core microbiome of healthy banana plants, a reduced OTU table was normalized at 19,892 reads. The core microbiome was defined as those OTUs at a dissimilarity level of 3% that were present in at least 50% of the respective samples. METAGENassist^51^ was employed to visualize co-occurrence patterns of taxa within the normalized core microbiome based on Spearman’s rank correlation. For visualizing relationships, core OTUs were summarized at genus level, and unassigned reads were excluded from the dataset.

## Additional Information

**How to cite this article:** Köberl, M. *et al*. Members of *Gammaproteobacteria* as indicator species of healthy banana plants on *Fusarium* wilt-infested fields in Central America. *Sci. Rep.* **7**, 45318; doi: 10.1038/srep45318 (2017).

**Publisher's note:** Springer Nature remains neutral with regard to jurisdictional claims in published maps and institutional affiliations.

## Supplementary Material

Supplementary Materials
