# Supplementary material for: Members of Gammaproteobacteria as indicator species of healthy banana plants on Fusarium wilt-infested fields in Central America
Source: Sci Rep. 2017 Mar 27;7:45318. doi: 10.1038/srep45318 (PMC5366900; doi:10.1038/srep45318)
Supplement: Supplementary Materials [file srep45318-s1.pdf]

Members of *Gammaproteobacteria* as indicator species of healthy banana plants on *Fusarium* wilt-infested fields in Central America

Martina Köberl[1], Miguel Dita[2,3], Alfonso Martinuz[3], Charles Staver[4], Gabriele Berg[1]

[1]*Graz University of Technology, Institute of Environmental Biotechnology, Austria*

[2]*Brazilian Agricultural Research Corporation – Embrapa, Brasília, Brazil*

[3]*Bioversity International, Turrialba, Costa Rica*

[4]*Bioversity International, Montpellier, France*

Table S1. Richness estimates and diversity indices for gammaproteobacterial 16S rRNA gene amplicon libraries at 3%, 5% and 10% genetic dissimilarity.

	Clusters[b] (OTUs)			Chao1 (OTUs)			Coverage (%)			Shannon (H')		
Sample[a]	3%	5%	10%	3%	5%	10%	3%	5%	10%	3%	5%	10%
N-S1-(1)	537	367	114	1,543	942	216	34.8	38.9	52.8	4.30	3.25	2.26
N-S1-(2)	618	420	128	1,940	1,054	214	31.9	39.8	59.5	4.01	3.29	2.29
N-S1+(1)	710	538	146	2,570	1,574	244	27.6	34.2	59.7	4.55	4.09	2.77
N-S1+(2)	654	441	119	2,030	1,230	221	32.2	35.9	53.7	4.12	3.09	2.04
N-S2-(1)	762	541	167	2,564	1,488	275	29.7	36.4	60.7	5.54	4.63	3.51
N-S2-(2)	694	535	172	2,301	1,443	304	30.2	37.1	56.5	4.82	4.18	3.03
N-S2+(1)	807	618	193	2,677	1,698	310	30.1	36.4	62.1	5.59	5.06	3.57
N-S2+(2)	770	561	164	2,266	1,350	279	34.0	41.5	58.8	5.63	4.94	3.49
N-S3-(1)	896	682	199	3,081	1,869	335	29.1	36.5	59.5	5.65	4.99	3.37
N-S3-(2)	1195	868	223	3,773	2,234	362	31.7	38.9	61.5	7.28	6.37	4.28
N-S3+(1)	893	634	174	3,080	1,809	298	29.0	35.1	58.5	5.47	4.60	3.04
N-S3+(2)	737	533	170	2,410	1,471	294	30.6	36.2	57.8	4.66	3.90	2.81
N-Re1-(1)	467	303	108	1,626	785	203	28.7	38.6	53.1	3.39	2.57	2.08
N-Re1-(2)	368	156	56	800	259	77	46.0	60.3	72.2	5.28	3.99	3.00
N-Re1+(1)	432	183	58	1,142	475	102	37.8	38.6	57.2	3.73	2.11	1.56
N-Re1+(2)	687	431	130	1,551	907	192	44.3	47.5	67.7	5.16	3.89	2.70
N-Re2-(1)	523	378	121	1,008	660	174	51.9	57.3	69.6	4.71	3.61	2.63
N-Re2-(2)	438	335	111	760	510	153	57.7	65.7	72.4	5.83	5.20	3.97
N-Re2+(1)	195	69	23	589	199	47	33.0	34.8	49.4	1.80	0.94	0.79
N-Re2+(2)	364	237	89	838	487	157	43.4	48.7	56.5	3.24	2.14	1.63
N-Re3-(1)	490	357	122	709	497	164	69.1	72.0	73.9	4.95	3.45	2.30
N-Re3-(2)	197	99	43	473	278	84	41.6	35.6	50.7	2.10	0.75	0.52
N-Re3+(1)	639	474	156	1,231	916	238	51.9	51.7	65.7	5.35	4.57	3.50
N-Re3+(2)	281	87	27	689	215	69	40.7	40.3	39.0	3.20	1.70	1.12
N-Ps1-(1)	58	41	11	122	56	14	47.2	73.4	80.7	1.55	1.41	0.51
N-Ps1-(2)	148	59	24	354	150	42	41.8	39.6	56.8	2.21	1.29	1.13
N-Ps1+(1)	205	58	12	601	114	20	34.2	50.8	59.9	2.81	1.77	1.52
N-Ps1+(2)	322	177	65	849	389	115	37.9	45.6	56.0	2.96	2.21	1.82
N-Ps2-(1)	238	77	24	512	146	31	46.5	53.0	75.1	2.90	1.82	1.66
N-Ps2-(2)	231	80	20	498	155	35	46.4	51.6	57.1	3.02	1.59	1.13
N-Ps2+(1)	195	57	15	391	108	27	49.8	53.0	55.4	2.74	1.41	1.13
N-Ps2+(2)	221	69	17	558	135	28	39.6	50.8	59.6	2.56	1.99	1.05
N-Ps3-(1)	248	109	37	511	222	51	48.5	49.3	72.4	2.98	1.68	1.30
N-Ps3-(2)	217	110	40	506	215	65	42.9	51.4	62.2	2.77	1.98	1.53
N-Ps3+(1)	265	81	18	668	176	35	39.6	46.1	51.0	2.88	1.50	1.10
N-Ps3+(2)	251	112	39	573	246	69	43.8	45.4	56.5	3.09	1.96	1.45
N-L1-(1)	168	72	15	502	128	25	33.5	56.4	58.6	2.63	2.11	1.19
N-L1-(2)	198	135	46	601	273	79	33.0	49.6	58.8	2.38	2.41	1.15
N-L1+(1)	120	45	20	365	118	32	32.7	38.1	63.2	0.84	0.42	0.34
N-L1+(2)	109	50	8	287	102	14	37.9	48.8	51.9	2.15	1.53	0.89
N-L2-(1)	227	76	16	534	138	20	42.5	55.2	80.4	2.67	1.35	1.04
N-L2-(2)	217	76	28	468	157	43	46.3	48.5	64.9	2.64	1.33	1.01
N-L2+(1)	206	57	13	514	106	18	40.0	53.6	71.4	2.45	1.49	1.09
N-L2+(2)	172	60	16	433	130	31	39.6	46.3	51.5	2.49	1.64	1.16
N-L3-(1)	284	131	43	778	287	67	36.5	45.8	64.3	3.50	2.31	1.95
N-L3-(2)	159	54	18	428	134	32	37.1	40.2	56.0	1.45	0.69	0.61
N-L3+(1)	403	191	67	1,071	416	106	37.6	45.9	63.0	3.90	2.39	1.76
N-L3+(2)	165	42	8	333	81	11	49.4	51.5	66.8	2.25	1.22	1.02
C-S1-(1)	209	139	52	485	242	83	43.1	57.6	63.1	4.86	4.74	3.75
C-S1-(2)	107	83	22	190	119	27	56.4	70.2	79.1	3.57	3.67	2.94
C-S1+(1)	177	86	18	389	136	31	45.5	63.3	58.4	2.16	2.01	1.23
C-S1+(2)	26	23	5	66	35	6	39.4	66.0	83.1	0.72	1.20	0.76
C-S2-(1)	67	44	11	123	60	15	54.4	73.8	73.2	2.11	2.17	1.91

C-S2-(2)	413	241	74	836	387	102	49.4	62.2	72.8	5.22	4.81	4.30
C-S2+(1)	95	39	10	197	77	19	48.1	50.6	51.6	1.95	2.01	1.54
C-S2+(2)	37	21	3	65	26	4	56.5	81.5	84.6	1.24	1.22	0.01
C-S3-(1)	186	141	29	363	247	45	51.4	56.9	65.6	3.89	3.89	3.38
C-S3-(2)	413	363	123	1,127	781	180	36.6	46.4	68.3	6.75	6.61	4.58
C-S3+(1)	157	103	48	304	178	71	51.8	57.9	67.5	4.37	4.27	3.18
C-S3+(2)	107	22	7	215	31	8	49.8	70.5	82.2	2.79	2.48	1.18
C-Re1-(1)	405	285	105	682	416	134	59.4	68.4	78.2	6.57	5.62	3.91
C-Re1-(2)	311	246	90	503	373	118	61.8	65.8	76.4	4.06	3.75	2.50
C-Re1+(1)	793	585	161	1,874	1,119	217	42.3	52.3	74.1	7.26	6.79	3.95
C-Re1+(2)	871	639	196	2,062	1,327	282	42.2	48.2	69.5	7.16	6.36	4.69
C-Re2-(1)	533	398	147	1,073	763	208	49.7	52.1	70.9	6.53	5.87	4.33
C-Re2-(2)	496	361	124	1,177	708	210	42.2	51.0	58.8	5.85	4.85	3.79
C-Re2+(1)	751	597	211	1,540	1,080	311	48.7	55.3	67.6	7.35	6.84	5.02
C-Re2+(2)	548	355	119	1,181	687	194	46.4	51.7	61.5	5.00	3.76	2.90
C-Re3-(1)	555	373	123	1,251	708	177	44.3	52.8	69.6	5.48	4.65	3.45
C-Re3-(2)	838	665	207	1,797	1,283	313	46.6	51.8	66.2	7.32	6.67	4.84
C-Re3+(1)	358	218	83	781	425	132	45.9	51.4	62.9	2.91	1.90	1.28
C-Re3+(2)	231	116	46	407	146	56	56.6	79.2	81.3	3.21	2.39	1.60
C-Ps1-(1)	107	35	15	230	76	25	46.3	46.6	60.2	3.53	2.42	2.15
C-Ps1-(2)	108	28	8	184	44	10	58.8	64.2	78.2	1.94	1.82	1.21
C-Ps1+(1)	116	30	9	243	50	12	47.8	59.8	80.5	2.06	1.77	0.58
C-Ps1+(2)	157	56	7	294	70	9	53.3	79.3	75.6	3.33	2.83	0.99
C-Ps2-(1)	262	79	12	585	130	17	44.8	61.2	71.8	2.92	1.95	1.35
C-Ps2-(2)	410	114	24	909	210	34	45.1	54.1	70.3	3.69	1.92	1.34
C-Ps2+(1)	135	48	11	280	68	17	48.4	70.5	62.9	2.93	2.68	1.33
C-Ps2+(2)	210	64	10	448	99	17	46.9	64.4	61.4	3.54	2.39	1.10
C-Ps3-(1)	264	68	16	494	120	23	53.5	56.1	68.1	3.30	1.85	1.39
C-Ps3-(2)	132	84	21	279	122	36	47.4	69.0	56.7	3.00	3.19	2.08
C-Ps3+(1)	150	52	13	270	58	13	55.5	89.2	97.5	2.64	1.58	0.91
C-Ps3+(2)	138	23	8	242	72	11	56.8	32.5	75.5	1.89	0.31	0.10
C-L1-(1)	246	100	22	543	200	34	45.3	50.1	65.1	4.17	3.03	2.38
C-L1-(2)	236	82	17	596	158	22	39.6	52.1	74.6	3.61	3.02	2.26
C-L1+(1)	109	30	12	225	51	14	48.6	58.2	85.0	0.97	0.38	0.39
C-L1+(2)	39	21	14	106	45	25	36.5	45.9	58.7	0.26	0.18	0.16
C-L2-(1)	167	21	12	250	59	17	66.6	35.3	70.8	1.48	0.26	0.24
C-L2-(2)	175	58	16	404	92	25	43.2	62.8	64.3	3.56	2.55	2.15
C-L2+(1)	215	57	11	497	136	12	43.3	41.5	90.1	3.53	2.45	1.96
C-L2+(2)	425	147	43	973	278	59	43.7	52.9	73.5	4.42	2.82	2.17
C-L3-(1)	228	92	22	474	157	36	48.1	58.8	60.2	4.08	3.30	2.43
C-L3-(2)	190	81	23	348	135	32	54.5	59.8	70.4	3.09	2.60	1.09
C-L3+(1)	56	28	10	137	43	11	40.8	64.0	92.2	1.08	0.99	0.77
C-L3+(2)	119	50	29	338	96	44	35.3	52.2	66.4	3.19	2.73	2.43

[a]Sample abbreviations indicate: (1) country (N- = Nicaragua, C- = Costa Rica), (2) microenvironment (S = rhizosphere soil, Re = endorhiza, Ps = pseudostem, L = leaves), (3) farm (1, 2, 3), (4) infestation with *Fusarium oxysporum* f. sp. *cubense* (- = healthy, + = infested), and (5) independent replicate sample (1, 2).

[b]averaged rarefaction curves are depicted in Fig. S1.

Table S2. Statistical comparisons of the gammaproteobacterial communities between healthy and FOC-infested banana plants.

	**Nicaragua**	**Costa Rica**	**Together**
**Rhizosphere**	0.234	0.090	0.138
**Endorhiza**	0.825	0.645	0.652
**Pseudostem**	0.349	0.813	0.788
**Leaves**	0.838	0.255	0.319

p values of individual microenvironments for pairwise comparisons between healthy and infested plants ascertained by adonis test based on jackknifed weighted UniFrac distances.

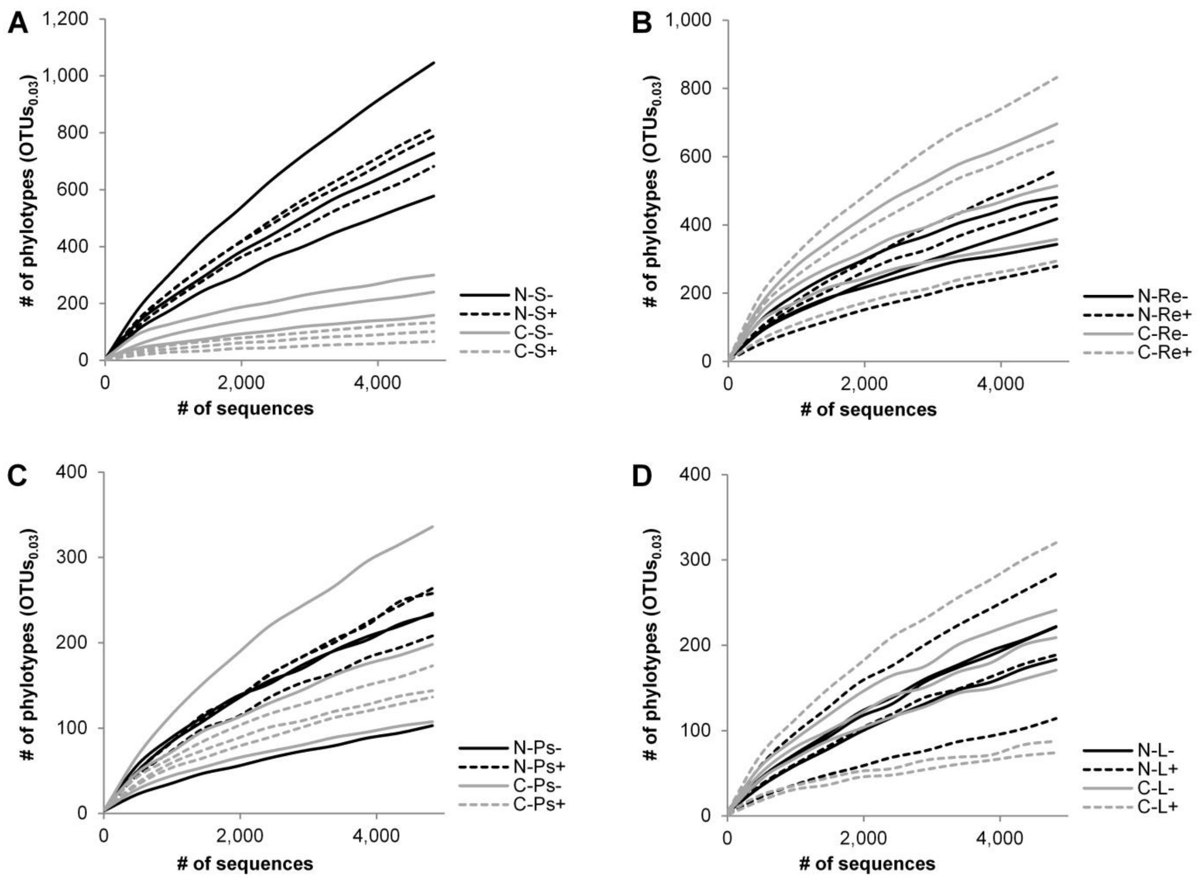

Figure S1. Rarefaction analysis of averaged *Gammaproteobacteria*-specific 16S rRNA gene sequence libraries at a dissimilarity level of 3% divided into rhizosphere soil (A), endorhiza (B), pseudostem (C), and foliage samples (D) and separated into samples from individual farms. N- = Nicaragua, C- = Costa Rica; S = rhizosphere soil, Re = endorhiza, Ps = pseudostem, L = leaves; - = healthy, + = *Fusarium* wilt-infested.

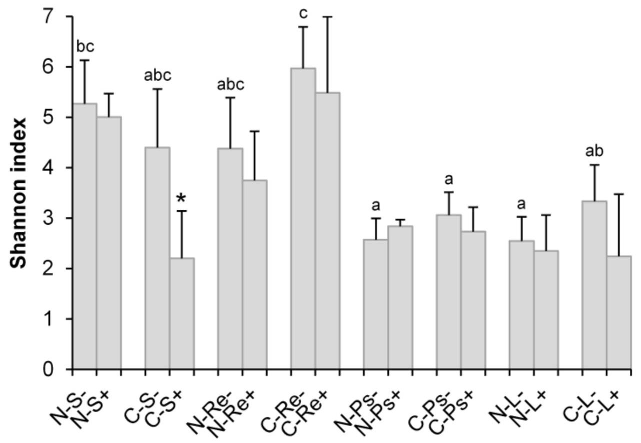

Figure S2. Shannon indices of gammaproteobacterial diversity for different banana-associated microenvironments divided into healthy and *Fusarium* wilt-infested state at a genetic distance of 3%, separated in samples from Nicaragua and Costa Rica. Data were ascertained by 16S rRNA gene profiling and are averages of the three sampling farms of a country ± confidence. N- = Nicaragua, C- = Costa Rica; S = rhizosphere soil, Re = endorhiza, Ps = pseudostem, L = leaves; - = healthy, + = infested. Significant differences ($p \leq 0.05$, Tukey-HSD post hoc test) between the microenvironments of asymptomatic plants are indicated by lowercase letters. Asterisks show significant differences ($p \leq 0.05$, independent samples *t*-test) between the healthy and infested state of the plant.

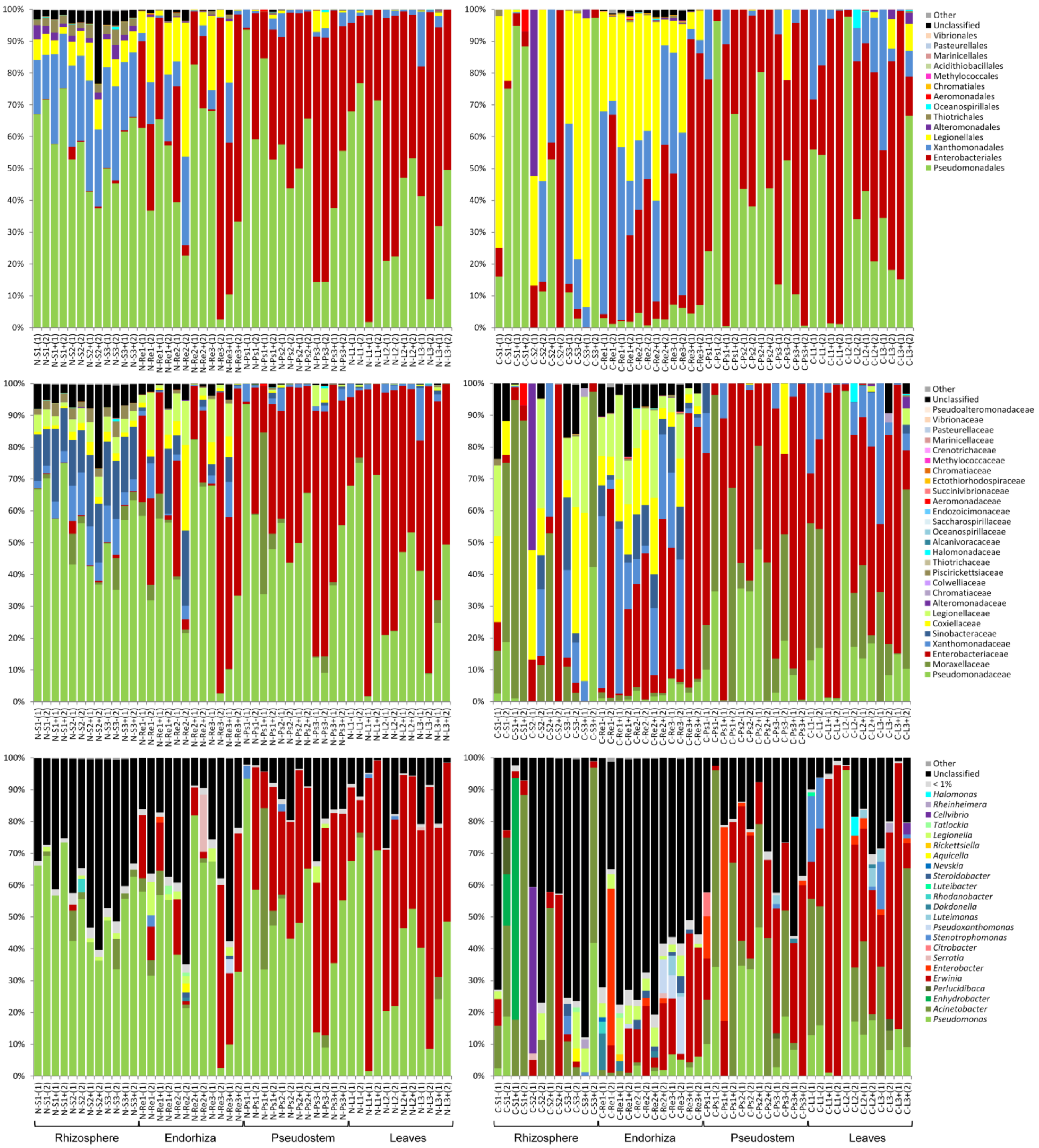

Figure S3. Taxonomic composition of *Gammaproteobacteria* communities inhabiting rhizosphere, endorhiza, pseudostem, and leaves of healthy and *Fusarium* wilt-infested banana plants grown in Nicaragua (left) and Costa Rica (right). Sequences obtained by Illumina MiSeq sequencing were classified at order, family and genus level. Samples were encoded using abbreviations indicating: (1) country (N- = Nicaragua, C- = Costa Rica), (2) microenvironment (S = rhizosphere soil, Re = endorhiza, Ps = pseudostem, L = leaves), (3) farm (1, 2, 3), (4) infestation with *Fusarium oxysporum* f. sp. *cubense* (- = healthy, + = infested), and (5) independent replicate sample (1, 2).
